# Multidirectional Planar Motion Transmission on a Single‐Motor Actuated Robot via Microscopic Galumphing

**DOI:** 10.1002/advs.202307738

**Published:** 2023-12-14

**Authors:** Lingqi Tang, Chenghao Wang, Songsong Ma, Yao Li, Bing Li

**Affiliations:** ^1^ School of Mechanical Engineering and Automation Harbin Institute of Technology Shenzhen 518055 China; ^2^ Guangdong Key Laboratory of Intelligent Morphing Mechanisms and Adaptive Robotics Harbin Institute of Technology Shenzhen 518055 China; ^3^ State Key Laboratory of Robotics and System Harbin Institute of Technology Harbin 150001 China

**Keywords:** crawling robot, eccentric rotation, galumphing motion, miniature robot, vibration actuation

## Abstract

Insect‐scale mobile robots can execute diverse arrays of tasks in confined spaces. Although most self‐contained crawling robots integrate multiple actuators to ensure high flexibility, the intricate actuators restrict their miniaturization. Conversely, robots with a single actuator lack the requisite agility and precision for planar movements. Herein, a novel eccentric rotation‐dependent multidirectional transmission is presented using a tilted eccentric motor and a simplistic two‐legged structural configuration for planar locomotion. The speed of the eccentric motor is modulated to enable alternating microscopic jumps to propel the system, creating a mode of motion analogous to galumphing of seals. Upon modeling the motion dynamics and conducting experiments, the effectiveness of direct motion transmission is substantiated through microscopic galumphing encompassing left/right crawling and straight‐forward crawling. Finally, a 1.2 g untethered robot is developed, which demonstrates enhanced straight crawling and spot turning, traverses narrow tunnels, and achieves precise movements. Therefore, the proposed motion‐transmission technique provides a comprehensive set of innovative solutions of underactuated agile robots.

## Graphical abstract

Planer locomotion can be actuated by a single eccentric motor via eccentric‐rotation‐dependent multidirectional transmission (ERDMT). Upon modeling the motion dynamics and conducting experiments, the effectiveness of direct motion transmission is substantiated through microscopic galumphing. Finally, a 1.2 g untethered robot is developed, which demonstrates enhanced straight crawling and spot turning, traverses narrow tunnels, and achieves precise movements.

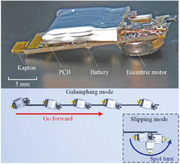

## 1 Introduction

Microrobotics is an emergent field focused on the development of small‐scale systems, with the objective of extending the functional scope of robotic applications. Specifically, micro crawling robots represent a prominent category within microrobotics and offer significant advantages for exploration in spatially constrained and narrow environments. However, miniaturization of the robotic structure and preservation of its flexibility are formidable challenges. For example, RoACH^[^ ^1^ ^]^ is distinguished by its smart composite microstructures (SCM) and dual‐shape memory alloy actuators. Although the robot is lightweight (2.3 g), the energy requirement for driving shape memory alloys is relatively high. Several piezoelectric actuators have been employed in HAMRs to improve the energy efficiency and motion agility, including the lightest 1.7 g hexapod robot HAMR^3^.^[^ ^2^ ^]^ Nonetheless, the deployment of discrete actuators restricts further miniaturization owing to the manufacturing constraints. In contrast, monolithic piezoelectric actuators have streamlined structural configurations. For instance, MinRAR utilized monolithic piezoelectric elements machined to actuate each bending actuator without mechanical joints, although this design necessitates complex high‐voltage excitation circuits.^[^ ^3^ ^]^ Kilobot^[^ ^4^ ^]^ is a representative robot actuated by two eccentric motors with reduced structure, simple excitation, and low cost. In general, crawling robots are composed of two or more actuators that promote in‐plane motion controllability. As such, a further reduction in the number of actuators can potentially hinder the mobility of the robot.

Recent studies have attempted to drive robots using a single actuator, because leveraging a single‐actuator architecture can simplify the structure and control technique. Nonetheless, such robots remain underactuated for planar motion, thereby necessitating the development of innovative transmission or excitation techniques. To emulate insect‐like agility in planar locomotion, capabilities for on‐the‐spot steering and forward motion are crucial. A hexapod robot, 1STAR^[^ ^5^ ^]^ actuated by a servo motor, was the first to achieve controlled in‐plane motion. However, its transmission mechanism was intricate, and the robot was unable to perform spot turns owing to the coupling between translational and steering motions. PISCES,^[^ ^6^, ^7^ ^]^ a piezoelectric‐actuated walking robot, can perform both forward and spot‐turning movements with different walking modes modulated by varying actuation frequencies. However, the miniaturization of PISCES is constrained by its complex high‐voltage excitation circuitry. Thus, incorporation of eccentric motors can result in more compact robots. For instance, Simobot^[^ ^8^ ^]^ simplified and miniaturized it using an eccentric motor that enabled it to execute turns with a small radius. Simobot can establish a forward trajectory by executing multiple small radius turns successively. However, this forward motion was not a product of direct motion transmission and thus demanded intricate path planning and excitation techniques. Currently, existing single‐actuator robots still have a range of limitations such as motion uncertainty, control challenges, and structural intricacies, which inhibit their applicability in future scenarios.

The planar motions of all micro crawling robots typically depend on the ground slipping of body components, actuated by either conventional active legs^[^ ^1^ ^]^ or vibrations.^[^ ^9^, ^10^ ^]^ Robots such as RoACH, HAMR, and 1STAR, which employ active leg‐based slipping, are self‐contained because of their efficient mechanisms. However, their transmission systems are intricate, and their crawling trajectories are imprecise.^[^ ^11^ ^]^ In contrast, vibration‐based slipping mechanisms (MinRAR, Kilobot, PISCES, and Simobot) offer stable motion transmission through simplified structures, although are multiple actuators are required for agile locomotion.

**Figure 1:**
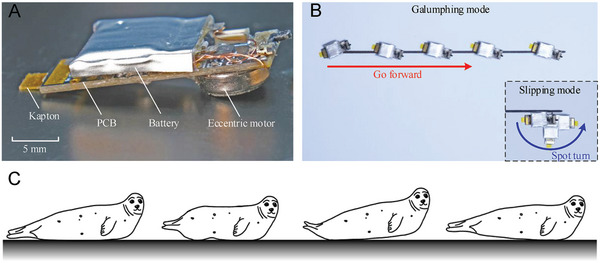
Single motor actuated crawling robot. A) GASR comprises a rigid body (PCB and battery), eccentric motor, and compliant hind leg (composed of Kapton), with all components bound together. The robot contacts the ground with the motor and hind legs. B) GASR can perform forward motion in galumphing mode and steering motion in slipping mode. C) A diagram of galumphing, which is characteristic gait of seals during terrestrial movement.

The planar movement of the robot is a distinct type of motion analogous to the galumphing of seals (as shown in **Figure** **1**C; Note S9, Supporting Information). Characterized by short front flippers and non‐rotatable rear flippers, seals traverse land through a belly‐wriggling motion that alternately lifts their fore and hind bodies off the ground. Compared to slipping, galumphing appears to be well‐suited for underactuated and miniaturized robots. Recent developments have demonstrated that robots utilize galumphing for forward movement. Piezoelectric soft robots^[^ ^12^, ^13^ ^]^ accomplish rapid galumphing (≈200 Hz) but suffer from motion instability owing to their flexible structures. Conversely, hopping robots,^[^ ^14^, ^15^, ^16^ ^]^ actuated by servomotors, can achieve stable and efficient galumphing at lower frequencies (<50 Hz). Nonetheless, prior implementations situated the actuator on the vertical plane, necessitating an additional module for horizontal steering. This dual‐actuator setup can result in asynchronous movements that impede the forward motion. Addressing this concern, the inclination of an eccentric motor has emerged as a potential solution because it generates both vertical and horizontal forces. However, inducing stable galumphing via an eccentric motor presents a formidable challenge, as leg‐bouncing motion is inherently unstable.^[^ ^9^, ^17^ ^]^ Furthermore, the issue of coordinating horizontal forces during stable galumphing remains unresolved, rendering the transmission of direct forward motion impossible. Therefore, a comprehensive understanding of galumphing motion and transmission, particularly when actuated by an eccentric motor, is yet to be achieved.

In this study, we introduce a novel vibration‐based galumphing motion‐transmission technique for single‐motor‐actuated microrobots. Utilizing an eccentric motor, the system achieved stable, microscale (30–400 µm) galumphing motions with precision. Notably, the prototype could execute both forward motion and lateral deflection solely through motor speed modulation, obviating the need for motor phase reversion. This phenomenon was identified as eccentric‐rotation‐dependent multidirectional transmission (ERDMT), establishing a direct methodology for planar motion transmission. Concurrently, the actuation of the eccentric motor enabled a combination of galumphing and slipping motions, thereby introducing spot‐turning capabilities for planar movements. A five‐degree‐of‐freedom (DOF) dynamic model was formulated to simulate these motions and elucidate the principles underlying the ERDMT. Subsequent validation of the dynamic model was conducted at both the micro and macro scales by capturing the actual movements of the prototype. Finally, we developed a 1.2‐g self‐contained galumphing and slipping robot (GASR, Figure 1) that precisely demonstrated spot turning and forward crawling. GASR revealed significant advantages in simplicity, agility, and precision, indicating promising prospects for exploration missions.

## 2 Results

### 2.1 Galumphing Motion

**Figure 2:**
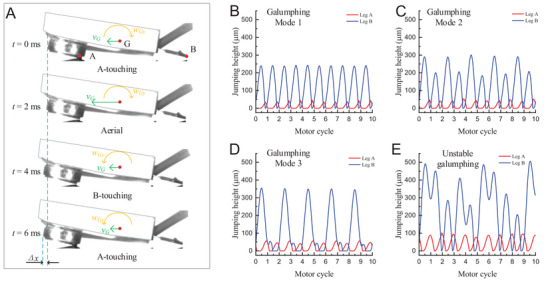
Observation and modeling results of galumphing motion. A) Observed motion sequences corresponding to galumphing mode 2 including three phases: A‐touching, aerial, and B‐touching. The period of motion corresponds to one motor cycle (6 ms). The forward velocity of the center of mass (COM) *v_G_* maintained positive, whereas the angular velocity of the body with respect to the *y*‐axis, *w_Gy,_* altered its direction. B) Mode 1 denotes a fundamental mode with a consistent galumphing height of both legs, and the jumping frequencies of (A) and (B) were the same as those of the motor period. C) In mode 2, the heights of leg B alternated high and low in a constant sequence, whereas the jumping frequency of A and B remained the same as motor frequency. D) In mode 3, leg B landed only once every two motor cycles. E) In unstable galumphing, leg B exhibited indeterminism jumping and leg A exhibited unstable toughing timing with the ground.

Forward galumphing motions were analyzed using high‐speed microscopic imaging. The prototypical motion sequence is shown in **Figure** **2**A. The robot initiated the A‐touching phase at 0 ms with leg A ascending into the air at 2 ms. Subsequently, leg B was in contact with the ground at 4 ms and reverted to the A‐touching phase at 6 ms. The maximum forward velocity, *v*_G_, occurred in the aerial phase, predominantly owing to the actuation of the rotor. Additionally, *v*_G_ remained positive throughout all phases owing to inertia, whereas the angular speed *w*_Gy_ around the *y*‐axis exhibited frequent directional changes. Each galumphing cycle was concluded with microscale forward displacement *Δx*.

Figure 2 outlines three specific galumphing modes based on the distinct motor speeds. Mode 1, illustrated in Figure 2B, was observed at low motor speeds ranging from 720–826 rad s^−1^. Here, legs A and B alternated the ground contact, achieving jumping peaks of 33 and 240 µm, respectively. As the motor speed increased (826–877 rad s^−1^, Figure 2C), the robot transitioned into galumphing mode 2, wherein the jumping peak alternated higher (289 µm) and lower (201 µm) in contiguous motor cycles. Finally, at a high motor speed (877–930 rad s^−1^, Figure 2D), leg B jumped higher (345 µm) in the first motor cycle and could only contact the ground in the second motor cycle. This phenomenon was attributed to the increased jumping height and reduced periodic time, resulting in an inadequate time another landing. Unstable galumphing motions ensued when the motor speeds exceeded 960 rad s^−1^, manifesting as erratic motion of leg B and inconsistent ground‐phase timing for leg A (Figure 2E). The modeling results of the galumphing modes were also observed in (Movie S1 and Figure S6, Supporting Information). The theoretical and practical jumping heights from the approximate calculation revealed a small deviation (<100 µm) that did not affect the validation of the ERDMT.

### 2.2 Planar Motions

The progressive forward motion of the robot was orchestrated according to a defined motion cycle. Consequently, rectilinear movement was achieved by nullifying the angular speed. Nonetheless, the robot predominantly deviated either to the left or right owing to a specific asymmetry factor. Influenced by an eccentric rotor, this factor can reverse the turning direction. Thus, the critical status demarcating left from right turning constitutes rectilinear motion. In this section, we examine both the intricate factors governing ERDMT and its behavioral tendencies.

#### 2.2.1 ERDMT in Microscale

The dynamics model replicates ERDMT, hinging on the variation in ground‐contact timing. During stable *x*–*y* plane motion, the angular velocity *w_G_* of the body becomes periodic in each microscopic motion cycle (two motor cycles), indicating that *w_G_* always reverts to its initial value at the end of each cycle. Consequently, the total angular momentum about the z‐axis is zero in each cycle. Given the motion pattern of the rotor, the angular momentum imparted by the motor is zero. Under this zero‐angular momentum condition, the angular momentum resulting from frictional forces must be similarly null.

**Figure 3:**
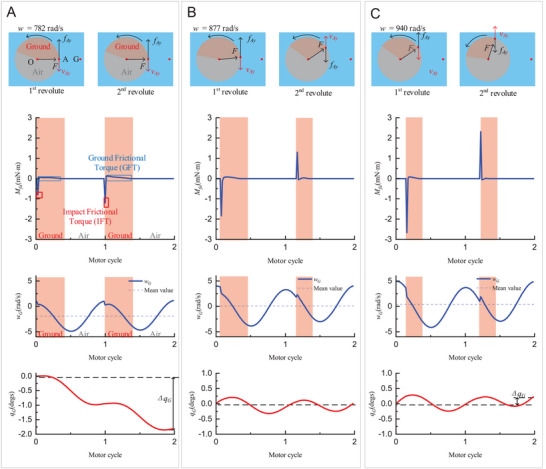
Driving principle of the ERDMT. The red‐shaded regions correspond to leg A‐touching the ground, whereas the gray‐shaded areas correspond to leg A jumping in air. When leg A lands, impact frictional torque (IFT) resists robot motion. In the following ground phase, the ground frictional torque (GFT) was similarly generated. In every two motor cycles, the contributions of the IFTs and GFTs entirely compensate each other to maintain the zero‐angular‐momentum condition. The frictional and motor contributions together determine the angular velocity *w_G_* of the body_,_ and their integration yields the rotation angle *Δq_G_*. The curve of *w_G_* varies with motor speed to satisfy the zero‐angular momentum condition. A) At a low motor speed (*w* = 782 rad s^−1^), both IFTs were negative, such that both GFTs were positive, indicating that the robot should rotate in the negative direction. Consequently, the mean value of *w_G_* was negative, and the robot rotated by a small negative angle *Δq_G_* in every motion cycle. B) At a critical motor speed (877 rad s^−1^), the ground phase of leg A changed, and a single IFT remained negative, whereas the other reverted to positive. The *w_G_* curve shifted up to a mean value of nearly zero. Therefore, the total rotation angle became nearly zero. C) When the motor speed further increased (920 rad s^−1^), the body angular velocity *w_G_* curve further shifted upward, resulting in a positive rotation angle.

Frictional contributions are explicitly outlined in **Figure** **3**. The red‐shaded regions denote periods when leg A makes ground contact, while the gray‐shaded regions signify airborne intervals for leg A. Upon leg A's contact with the ground, the generated impact frictional force *f_Ay_* gives rise to impact frictional torque (IFT). Likewise, sustained ground contact produces a ground frictional torque (GFT). The directional attributes of the frictional torques were determined by the actual velocity of leg A in the ground co‐ordinate system. Thus, these frictional contributions are modulated by the actual speeds and further influenced by *w_G_*. Note that the contributions from leg B were considered negligible.

The specificities of the ground‐contact timing can affect the *w_G_* curve because the zero‐angular momentum condition requires a specific frictional force direction. At the motor speed *w* of 782 rad s^−1^ (Figure 3A), both IFTs were negative owing to the positive rotational direction relative to the COM when landing. Thereafter, the GFTs must provide equal but opposing momentum to counterbalance the IFT contributions. This resulted in a predominantly negative rotation during the subsequent ground phase, rendering the mean value of *w_G_* negative (Figure 3A). Similarly, the rotation angle of each cycle *Δq_G_* is also negative. Given that the robot operates under an undamped forced oscillation, the phase position of *w_G_* is contingent on the phase position of the excitation (motor force). In other words, *w_G_* is maximized whenever the rotor returned to its initial position, facilitated by the integer moment from the prior motor cycle. Thus, variations in the motor speed can only induce vertical shifts in the *w_G_* curve to satisfy the zero‐momentum condition.

Different ground‐contact timings result in a unique y‐positioning of the *w_G_* curve. Similarly, when the motor speed increased to 877 rad s^−1^ (Figure 3B), the initial IFT was negative but became positive in the subsequent landing. Thus, the value of *w_G_* should remain mostly transversely symmetric in the ground phases owing to the directional demand of the GFT. Consequently, the rotation angle *Δq_G_* was approximately zero. However, when the motor speed increased to 940 rad s^−1^, the ground‐touching timing varied, causing the *w_G_* curve to shift upward, such that the mean value of *w_G_* was positive. Thus, rotation angle *Δq_G_* was positive.

#### 2.2.2 ERDMT in Macroscale

**Figure 4:**
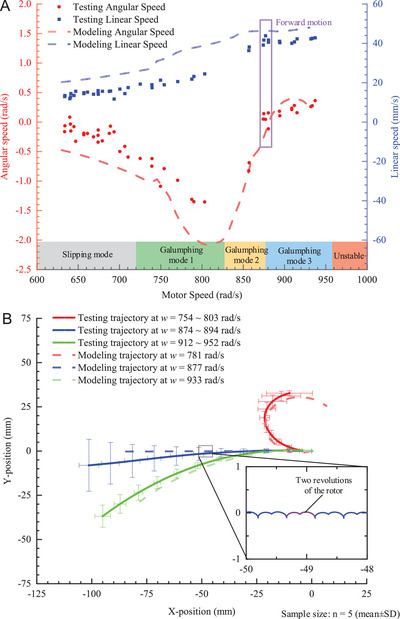
Crawling trajectories induced by ERDMT. A) Evaluation of angular velocities versus motor speeds. At lower motor speed, the robot stick‐slipped on the ground and began galumphing as the motor speed increased. Zero‐angular speed galumphing appeared at ≈870 rad s^−1^. B) Comparison of modeling and testing trajectories. The dashed and solid lines represent the modeling and testing trajectories, respectively. Magnified subplots displayed the microscopic movements of COM in the *x*–*y* plane, and the purple curvatures represent the trajectory in two motor cycles.

ERDMT induced various planar trajectories under varying motor speeds. In particular, *w*_Gtest_, *v*_Gtest_, *w*_Gmodel_, and *v*_Gmodel_ are defined as the mean body angular and translational speeds of the COM in testing and modeling, respectively. In **Figure** **4**A, during stable motion periods (2.5 s) at constant motor speeds, as the motor speed increased during various tests, the amplitude of the *w*_Gtest_ increased to −1.25 rad s^−1^ but later decreased. As the motor speed sequentially increased, *w*_Gtest_ reversed its direction at ≈870 rad s^−1^ and increased to ≈0.5 rad s^−1^ at ≈930 rad s^−1^. Overall, the modeling results revealed a similar trend. When the motor rotated at 630 rad s^−1^, the mean *w*_Gmodel_ was −0.38 rad s^−1^, and *w*_Gmodel_ decreased to −2.27 rad s^−1^ as motor speed increased to 835 rad s^−1^. Thereafter, the amplitude of the *w*_Gmodel_ returned to 0 at a speed of 877 rad s^−1^. Subsequently, *w*_Gmodel_ became positive and increased slightly to 0.45 rad s^−1^. The modeling and testing results revealed a highly consistent tendency. As the motor speed increased in the same scope, the linear speed *v_Gtest_* increased from 11.9 to 43.9 mm s^−1^, whereas the linear speed *v*_Gmodel_ increased from 20 to 48 mm s^−1^.

The ERDMT was validated based on both computational modeling and experimental testing. Minor discrepancies between the two sets of results could arise from variations in the parameter settings or approximate calculations. The inversion of the rotational direction occurred at a motor speed of 877 rad s^−1^. At this pivotal speed, the robot exhibited zero‐angular velocity, traversing a straight trajectory (purple box in Figure 4A). Moreover, in galumphing mode 1, the amplitude of *w*_Gmodel_ primarily increased, the value of *w*_Gmodel_ decreased in galumphing mode 2, and the critical speed of ERDMT was close to the boundary between galumphing modes 2 and 3.

The prototype robot demonstrated capabilities for straight, left, and right crawling motions, illustrated in Figure 4B. Within a motor speed range of 754–803 rad s^−1^, the robot veered to the right with an average radius of 18.5 mm and a mean speed of 21.8 mm s^−1^. Conversely, at higher motor speeds ranging from 912 to 952 rad s^−1^, the robot deviated to the left, registering a radius of 130.4 mm and speed of 40.3 mm s^−1^. A distinct speed window spanning 874–894 rad s^−1^ enabled a near‐linear motion with an average speed of 41.2 mm s^−1^. The root mean square errors for the test trajectories during right, left, and straight crawling were 8.99, 9.81, and 8.53 mm, respectively, when compared to the computational models. Notably, the actual straight crawling trajectories exhibited a mean straightness error of 4.14 mm, whereas the modeled trajectories displayed a significantly lower straightness error of only 0.09 mm. This disparity can be primarily attributed to motor speed fluctuations at a constant voltage, averaging at ±28.4 rad s^−1^.

### 2.3 GASR: Performance Experiments and Evaluation

GASR is a miniature crawling robot actuated by a single eccentric motor and was developed by incorporating galumphing and slipping motions. The robot employs a model‐based design methodology, as described in Note S4 (Supporting Information). By merely comprising four components, as illustrated in Figure 1, GASR can execute both forward and steering movements under variable driving voltages. These voltages were modulated by the PWM output of a PCB. The 25‐mm‐long robot weighing only 1.2 g is self‐powered and remote‐controlled via Bluetooth.

**Figure 5:**
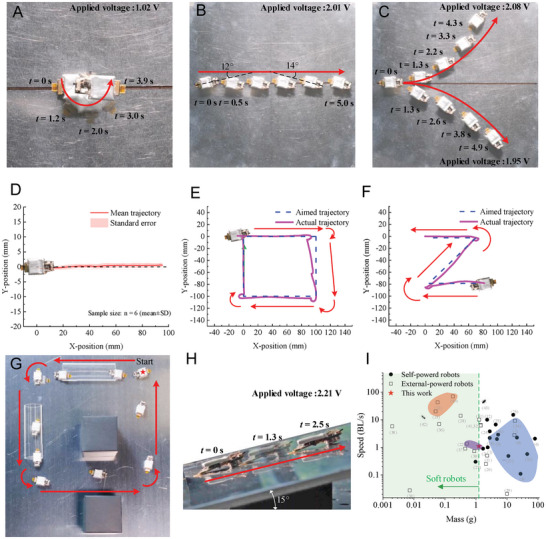
Performance of a miniature and agile robot GASR. A) With a low input voltage, the GASR steered in place at 45°/s. B) With 2.01 V input, GASR crawled straight forward at 1.1 BL s^−1^. C) By slightly increasing or decreasing the input voltages with respect to that of straight moving, GASR exhibited left or right crawling, respectively. D) GASR revealed high trajectory precision in the repetitive test (*n* = 6 repeats; foam board substrate). GASR crawled following a *z*‐shaped path E) and a square path (F). G) GASR crawled in a loop with two narrow tunnels. H) GASR could climb a 15°slope in 28.1 mm s^−1^ with 2.21 V input voltage. I) Maximum crawling speeds versus mass of robots. Robots in the red region perform unstable galumphing, whereas those in the blue region perform vibration‐based slipping. The purple region refers to robots with vibration‐based slipping and stable galumphing locomotion.

GASR operates in three distinct modes: straightforward crawling, forward left/right crawling, and on‐the‐spot turning. In **Figure** **5**A, under an applied voltage of 1.02 V, the robot steered counterclockwise at a rate of 45°/s. Conversely, upon applying a voltage of −1.02 V, clockwise steering was observed at −40°/s (Movie S2, Supporting Information). The forward motion (Figure 5B) was generated under 2.01 V, with a mean speed of 28.3 mm s^−1^, corresponding to 1.1 body length (BL). The robot initiated motion with a minor deflection angle of 12° and self‐misalignment angle of 14° relative to its direction of movement. At a reduced actuation voltage of 1.95 V, the robot executed a clockwise circular trajectory characterized by an angular speed of 10°/s and a radius of 232 mm. Moreover, under a voltage of 2.08 V, a counterclockwise circular motion was observed, featuring an angular speed of 11°/s and a radius of 143 mm. Additionally, GASR demonstrated stable, straight‐line crawling on both firm (aluminum board) and soft substrates, as viewed in Movie S2 (Supporting Information). A repetitive test on foam board indicated a mean lateral error across six trials of 1.1 ± 0.35 mm, or 1.2% (Figure 5D).

GASR manifests both agility and precision in various mimetic applications. Figure 5E,F depicts the robot's proficiency in the following square and “Z”‐shaped trajectories. It accomplished continuous straight and turning movements to follow the desired paths with minor straightness errors (<6 mm) and turning radii (<5 mm), completing each path in 29 and 30 s, respectively. Figure 5G and Movie S3 (Supporting Information) show the capability of GASR in traversing tunnels; it navigated through a 25‐mm‐wide tunnel, executed a spot turn to proceed through another tunnel, and returned to its original position. Furthermore, the robot was competent in ascending slopes. On a 15° incline, as shown in Figure 5H, GASR maintained a comparable straight‐line speed of 28.1 mm s^−1^ by slightly elevating the applied voltage to 2.21 V. The maximum uphill crawling angle is 22°, while the maximum downhill crawling angle is 30°. Additionally, the robot can currently function well on surface with mild roughness (P320 Grade sandpaper, aluminum plate, foam board, etc.). Details can be found in Note S10 (Supporting Information).

## 3 Discussion

Stable galumphing motion can be achieved over a broad range to the proper structural design. The eccentric motor, oriented with its output in the vertical plane, generates varying vertical forces that actuate the galumphing movement. The prototype exhibits a broad, stable galumphing range, and its stability is grounded in three key elements: i) The strategic placement of the motor at the bottom of the structure creates a small tilt angle relative to the ground; ii) The squared shape of the robot body extends the distance between the two legs, consequently minimizing instability due to pitch angle fluctuations; and iii) Compliant legs mitigate unexpected vibrations, whereas a rigid main body maintains a stable natural frequency. When periodic galumphing was achieved, the system remained stable and robust, even when subjected to input fluctuations (±28.4 rad s^−1^). In contrast, soft robots, with their inherently variable forms, encounter difficulties in achieving stable galumphing. Furthermore, the motion becomes unstable if the vibration absorbers are removed, as rigid legs tend to cause high jumps, leading to unstable galumphing. Nonetheless, configurations exist, such as those employing a rigid hind leg, enabling both low jumps and stable movements, as shown in Figure S8 (Supporting Information).

The ERDMT‐based galumphing motion is advantageous in terms of motion complexity. Unlike a previous study^[^ ^18^ ^]^ that reported unpredictable movements, we developed a theoretically predictable prototype capable of straightforward crawling, in accordance with the ERDMT model. The prototype could follow a straight line with minimal error, which was likely attributable to motor instability and ground unevenness. Specifically, the overall trajectory was influenced by fluctuations in motor speed at critical velocities. This critical speed for the ERDMT model is situated near the transitional boundary between galumphing modes 2 and 3. Variations in speed can give rise to asymmetric crawling paths. Notably, the rotation angles change more swiftly when the motor speeds decrease, as illustrated in Figure 4. This is because lower motor speeds result in two negative impact frictional torques, whereas higher speeds produce one negative and one positive torque. Consequently, the overall rotational speed of the robot decreases rapidly with decreasing motor speeds. Additionally, the implementation of galumphing ensures that the microscale trajectory did not contain backward components, in contrast to conventional eccentric motor robots that rely on stick‐slip mechanisms.^[^ ^9^ ^]^ This demonstrates greater efficiency, as the COM maintains a forward velocity throughout the movement.

Drawing on the galumphing and slipping motions, we propose a lightweight multimodal crawling robot. Within the realm of small‐scale planar crawlers, the key performance metrics include mass, speed, and power source, as depicted in Figure 5I.^1^, ^2^, ^3^, ^4^, ^5^, ^6^, ^7^, ^8^, ^10^, ^11^, ^12^, ^13^, ^17^, ^19^, ^22^, ^23^, ^24^, ^25^, ^26^, ^27^, ^28^, ^29^, ^30^, ^31^, ^32^, ^33^, ^34^, ^35^, ^36^, ^37^, ^38^, ^39^, ^40^, ^41^, ^42^, ^43^, ^44^ Soft‐crawling robots are predominantly externally powered and can weigh as little as 0.9 g.^[^ ^19^ ^]^ Conversely, many rigid crawling robots utilize battery power and can weigh as little as 1.7 g.^[^ ^2^ ^]^ The GASR possesses a light weight of 1.2 g and an average speed of 1.1 BL s^−1^, nearly rivaling externally powered robots. Furthermore, robots employing unstable galumphing and vibration‐based slipping are located within the red and blue regions of the performance space, respectively. Robots that utilize galumphing are characterized by high speed and structural simplicity. However, issues such as unstable or asynchronous galumphing can impair forward motion in two‐module configurations. Conversely, vibration‐based slipping robots benefit from simple structures and stable motions that are attributed to the underlying slipping principle. In this study, we amalgamate stable galumphing and vibration‐based slipping into a multimodal robot capable of both stable forward movement and effective steering.

GASR revealed high agility and simple excitation among existing small‐scale crawling robots. Meanwhile, the weight and power consumption were also advanced among untethered robots (see details in Table S4, Supporting Information). The robot incorporates advantageous elements from both eccentric rotating drum multi trajectory and multimodal locomotion, resulting in a structurally simple and cost effective design ($3.7). The required excitation is uncomplicated, necessitating only a one‐way low‐voltage input (<3 V). Moreover, ERDMT is a universal model that is easily replicable using common materials, as elaborated in Note S5 (Supporting Information). Additional benefits of vibration‐based actuation include stable and precise movement, even without feedback control. This results in a significantly reduced trajectory error compared to conventional legged crawling robots.^[^ ^11^ ^]^ Although single‐motor actuation typically exhibits less agility and is generally deficient in spot‐turning capabilities, we successfully achieved both spot‐turning and straight crawling through multimodal locomotion. Consequently, the ERDMT effectively expands the repertoire of motion principles available for crawling robots.

## 4 Materials and Methods

### 4.1 Structural Configuration

**Figure 6:**
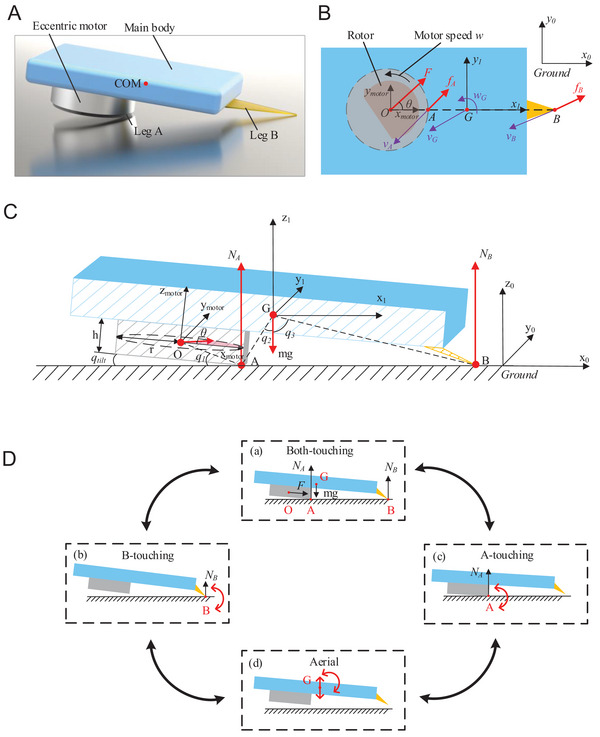
Schematic of the dynamics model. A) Illustrative 3D model of the structural configuration. Schematic of the dynamics model in B) top view and C) side view (section along the x–z plane). D) Galumphing motions in the x_0_–z_0_ plane consisting of four phases: (a) Both‐touching: both legs stand on the ground; (b) B‐touching: only leg B stands on the ground, and the robot swings around leg B; (c) A‐touching: only leg A stands on the ground; (d) Aerial: both legs are above the ground, and the robot's motion is an overlay of rotation around the COM (point G) and translation along the *z*‐axis.

In contrast to previous studies,^[^ ^9^, ^20^ ^]^ that positioned eccentric motors vertically for stable actuation and utilized dual‐motor configurations for steering, the present study employs a novel single‐motor configuration to significantly reduce the physical dimensions of the robot. In this underactuated system, an eccentric motor is strategically placed at a tilt angle to exert forces on both the vertical and horizontal planes. As depicted in **Figure** **6**A, the motor was installed in the lower section of the robot. Inspired by seal locomotion that necessitates coordinated movements of the fore and hind bodies, we carefully designed two distinct legs: Leg A incorporates the tilted motor, whereas leg B comprises a compliant Kapton plate situated at the rear end of the robot. These legs alternate in contact with the ground during galumphing gait.

Addressing the challenges associated with modeling, most vibration‐based slipping robots are equipped with circular bodies and tri‐leg configurations. While aiming for a geometric center as the COM, they often fail to achieve this ideal configuration owing to the errors induced during fabrication and assembly. In the current design, the circular outer shape of the robot, which is attributable to the motor, provides a self‐aligning mechanism through the tumbler effect. Consequently, the COM consistently resides in the longitudinal mid‐perpendicular plane, thereby mitigating the modeling errors.

According to the dynamic model, the design parameters can significantly affect the movement. The ratio between the robot's weight and the actuation is crucial. The motor output force should match the robot's weight in a specific range; otherwise, a larger motor output force would lead to unstable motion, and a smaller output would not be able to actuate ERDMT. Since the motor speed varies from ≈600 to ≈1000 rad s^−1^, the critical motor speed for straight‐motion transmission should lie in the middle of this range. Thus, the ratio was designed 3.5 for the best performance of ERDMT (see Notes S7 and S8, Supporting Information).

### 4.2 Modeling Overview

Three Cartesian co‐ordinate systems were constructed (Figure 6B,C), namely, the motor co‐ordinate system *x*_motor_ *, y*_motor_ *, z*_motor_, body‐co‐ordinate system *x_1_, y_1_, z_1,_* and ground co‐ordinate system *x*_0_ *, y*_0_ *, z*_0_ *_,_* respectively. The planar motion of the robot exhibits three DOFs, and considering the galumphing motions in the *x_0_–z_0_* plane, the dynamic model should allow translational motion in the *z_0_*‐axis and rotation around the *y_1_*‐axis. The five‐DOF dynamics model was implemented by modeling the two‐DOF galumphing motion (in vertical plane) and three‐DOF planar motion (in horizontal plane). We assumed that the eccentric motor was spinning at a constant speed *w* with zero output torque. To obtain the output force *F*, previous studies^[^ ^8^, ^21^ ^]^ applied the centrifugal force equation and neglected the internal frictional contributions. To improve the modeling accuracy, we measured the output force using an experimental‐based lookup table.

When the output force *F* of the motor is sufficiently large to elevate the robot in air, it can galumph. Unlike previous soft robots that galumph unstably,^[^ ^12^, ^13^ ^]^ GASR can perform regular and predictable galumphing. As depicted in Figure 6D, four phases of motion are involved in a complete galumphing cycle: both‐touching, A‐touching, B‐touching, and aerial. The dynamic parameters of each phase were sequentially solved and are detailed in Note S1 (Supporting Information).

### 4.3 Model of Galumphing Motion

The robot initiates its motion with the both‐touching phase, where both leg A and leg B are in contact with the ground. Assuming that the eccentric mass occupies position *θ* at time *t*, the output force *F* of the motor can be expressed as $\vec{F^{1}}$ in the body‐co‐ordinate system. Thus, the torques induced with respect to the mass center G $M_{G}^{1}$, leg A $M_{Ay}^{1}$, and leg B $M_{By}^{1}$ can be obtained. Suppose the vertical component of F is $F_{z}^{1}$, leg A/B jumps into the air when any of $F_{z}^{1}$, $M_{Ay}^{1}$ or $M_{By}^{1}$ is adequately large, and the robot performs a swing motion around the supporting leg (A‐touching/B‐touching phase). If both legs jump off the ground, the robot moves freely in the air (aerial phase), but lands on the ground within a few moments. These phases would occur in certain sequences and form periodic galumphing motions.

Landing collision causes an unsmooth motion transition. For instance, we present the collision process involving leg A. For the transient state before and after collision, we assume that leg A exhibits velocities *v_Az_* and $v_{Az}^{\prime }$, whereas COM exhibits velocities *v_Gz_* and $v_{Gz}^{\prime }$ as well as angular velocities *w_y_* and $w_{y}^{\prime }$, respectively. Assume the collision recovery coefficient is *e* according to the classical collision formula, we can obtain

(1) $$v_{Az}^{\prime }=-ev_{Az}$$

As only one force *N_A_* acts on leg A with *mg* is neglectable in the collision. Thus, according to the law of conservation of angular momentum around leg A, we can derive

(2) $$w_{y}I_{Gy}-mx_{AG}v_{Gz}=w_{y}^{\prime }I_{Gy}-mx_{AG}v_{Gz}^{\prime }$$

Thereafter, we can obtain $w_{y}^{\prime }$ and $v_{Gz}^{\prime }$. Assume the collision duration is *Δt*, we can obtain the impact force *N_A_* during collision based on momentum conservation in the *z*‐axis:

(3) $$N_{A}=\frac{m\left(v_{Gz}^{\prime }-v_{Gz}\right)}{\Delta t}$$

The action of leg A could be rebound or touchdown depending on the speed of leg A after collision. Thereafter, the motion status of the robot can be decided (detailed in Figure S2, Supporting Information).

### 4.4 Model of Planar Motion

The iterative method is favored for modeling due to its capability to handle diverse phase sequences, irrespective of their stability. In the context of galumphing motion, the timing and magnitude of ground forces *N_A_* and *N_B_*, as well as the frictional forces *f_A_* and *f_B_*, can be altered by the centrifugal forces. Owing to the synergy between motor and frictional forces, the robot's planar motion can be obtained. Assuming that the robot has rotated an angle *q_G_* in the *z*‐axis at time *t*, the corresponding rotation matrix $R_{1}^{0}$ can be derived by transforming the body‐co‐ordinate system to the ground co‐ordinate system.

(4) $$R_{1}^{0}=\left(\begin{array}{ccc} \cos q_{G} & -\sin q_{G} & 0\\ \sin q_{G} & \cos q_{G} & 0\\ 0 & 0 & 1 \end{array}\right)$$

To obtain the magnitudes and directions of frictional forces $\vec{f_{A}^{0}}$ and $\vec{f_{B}^{0}}$, the actual velocities of the two legs in the ground co‐ordinate system $\vec{v_{A}^{0}}$ and $\vec{v_{B}^{0}}$ should be initially obtained via the velocity and angular velocity of COM. Accordingly, the frictional force $\vec{f_{A}^{0}}$ can be obtained as follows:

(5) $$\vec{f_{A}^{0}}=\left\{\begin{array}{cc} -\frac{\vec{v_{A}^{0}}}{\left|\vec{v_{A}^{0}}\right|}u_{A}N_{A} & when\;\vec{v_{A}^{0}}\neq 0\\ -\frac{\vec{F_{m}^{0}}}{\left|\vec{F_{m}^{0}}\right|}u_{A}N_{A} & when\;\vec{v_{A}^{0}}=0\;\mathrm{and}\;\left|\vec{F_{m}^{0}}\right|>u_{A}N_{A}+u_{B}N_{B}\\ \mathrm{Not}\;\mathrm{applicable}\; & when\;\vec{v_{A}^{0}}=0\;\mathrm{and}\;\left|\vec{F_{m}^{0}}\right|\leq u_{A}N_{A}+u_{B}N_{B} \end{array}\right.$$

where $\vec{F_{m}^{0}}$ denotes the motor force in the ground co‐ordinate system. The frictional force $\vec{f_{B}^{0}}$ can be obtained similarly, and the acceleration of COM in the ground co‐ordinate system $\vec{a_{G}^{0}}$ can be derived as:

(6) $$\vec{a_{G}^{0}}=\left\{\begin{array}{cc} \frac{1}{m}\left(\vec{f_{A}^{0}}+\vec{f_{B}^{0}}+\vec{F_{m}^{0}}\right) & others\\ 0 & when\;\vec{v_{G}^{0}}=0\;\mathrm{and}\;\left|\vec{f_{A}^{0}}\right|+\left|\vec{f_{B}^{0}}\right|>\left|\vec{F_{m}^{0}}\right| \end{array}\right.$$

To obtain the angular acceleration $\vec{\beta_{Gz}^{1}}$, the overall torque $\vec{M_{G}^{1}}$ acting on COM in the body‐co‐ordinate system can be readily deduced as follows:

(7) $$\vec{M_{G}^{1}}=\vec{GA}\times \left(R_{0}^{1}\vec{f_{A}^{0}}\right)+\vec{GB}\times \left(R_{0}^{1}\vec{f_{B}^{0}}\right)+\vec{GO}\times \vec{F_{m}^{1}}$$

(8) $$\vec{\beta_{Gz}^{1}}=\frac{\vec{M_{G}^{1}}}{I_{Gz}}$$

where *I_Gz_* denotes the rotational inertia around the *z*‐axis at COM. Finally, we can obtain the motion parameters in the ground co‐ordinate by the iterative algorithm, namely the position of COM $\vec{P_{G}^{0}}$ and the angle of COM along the *z*‐axis $\vec{q_{Gz}^{1}}$:

(9) $$\left\{\begin{array}{c} \vec{P_{G}^{0}}=\;\int \int \;\vec{a_{G}^{1}}dt^{2}\\ \vec{q_{Gz}^{1}}=\;\int \int \;\vec{\beta_{Gz}^{1}}dt^{2} \end{array}\right.$$

## 5 Conclusion and Future Works

In this study, we advance the field of insect‐scale crawling robots by introducing a novel effect, namely ERDMT. Utilizing a single eccentric motor, our prototype robot induced a seal‐like galumphing motion under the principles of the ERDMT. Through rigorous dynamic analysis and empirical verification, the robot was found to be capable of transmitting motion in varying directions by modulating the motor speed. Moreover, the robot exhibited stable straight‐line crawling facilitated by a straightforward PWM scheme, thereby simplifying the circuit architecture. Consequently, we developed an untethered GASR with a weight of only 1.2 g, incorporating both straight galumphing and spot‐turning capabilities. Although microrobots often necessitate intricate and laborious fabrication processes, the GASR circumvented a majority of these limitations through its simplified two‐legged structural design. The operational mechanisms and architecture of GASR displayed remarkable versatility in planar motion, tunnel traversal, and slope climbing.

This research represents an inaugural exploration into this new motion paradigm. Future research is expected to focus on enhancing the feedback trajectory control of the robot through mechanisms of self‐perception and adaptation, along with the fine‐tuning of speed variables. Additionally, this rudimentary structural design offers the potential for further miniaturization by customizing motors and could feasibly be scaled up for navigation across diverse and challenging terrains. Owing to its rigid body and uncomplicated actuation, ERDMT‐based motion can be integrated with other systems. This establishes promising avenues for future multifunctional devices. For example, the vibrational functionality of mobile phones could be repurposed for motion control, thereby enabling capabilities such as fault detection and target tracking.

## 6 Experimental Section

### Motor Speed Measurement

To accurately validate the dynamics model, the actual motor speed must be recorded. Thus, the signal from both poles of the DC brushed motor (Figure S3B, Supporting Information) was measured with an oscilloscope (DSOX1204G‐200Mhz, Keysight; Figure S5, Supporting Information) at 10 kHz, which was ≈60 times the maximum frequency of the motor. Thereafter, the peaks produced by switching the brush were recorded. Accordingly, the motor speed could be calculated accurately using the equation: ω_*motor*_ = 0.5π*f_os_*, where ω_*motor*_ refers to the motor speed and *f_os_* refers to the frequency of the peaks recorded by the oscilloscope. The actual rotational motion of the rotor at 10 kHz was directly recorded using a high‐speed camera (Ispeed‐221 mono, Hadland). The results revealed that the oscilloscope‐based measurement method exhibited a mean error of 1.07% with respect to the observed speed. The output force test of the motor is in Note S2 (Supporting Information).

### Galumphing Motion Observation

Galumphing motions occur at the frequency and microscopic dimensions of the motor (30–500 µm). A high‐speed camera (Ispeed‐221 mono, Hadland) with a standard lens was used to record a complete view of the galumphing motion beyond 1000 Hz. For a close‐up perspective, the same camera with a 26‐mm f4 microlens was used from Kuangrenweiyan, Taobao. Notably, the robot must be illuminated using high‐power light (EF‐200, >23 000 Lm, Jinbei). The motor speed was measured using the aforementioned method.

### Planar Motion Test

To validate the modeling results, the planar motions of the robot were observed under various supply voltages from 1.65–2.50 V in increments of 0.05 V, which were repeated three times for each voltage. A DC power supplier (UTP1306S, UNI‐T) and the same oscilloscope was adopted to measure the motor speed, and the motion of the robot was observed using a motion‐capture system (VICON Vantage V5, Logemas; accuracy: 0.1 mm) at 200 Hz. The motor speed was measured using the aforementioned method, and the details of the experimental setup are presented in Note S3 (Supporting Information).

### Statistical Analysis

The sample size (n) for each statistical analysis was *n* = 3. The data were expressed as mean ± SD (Standard Deviation). Statistical analysis of the data was performed using OriginPro 2021.

## Conflict of Interest

The authors declare no conflict of interest.

## Author Contributions

L.T. and C.W. contributed equally to this work. Y.L. and L.T. performed conceptualization. Y.L. and L.T. performed the methodology. L.T. and C.W. performed investigation. L.T. and C.W. performed visualization. Y.L. and B.L. performed supervision. Y.L. and L.T. wrote the original draft. Y.L., L.T., C.W., S.M., and B.L. wrote the review and contributed to editing.

## Supporting information

Supporting Information

Supplemental Movie 1

Supplemental Movie 2

Supplemental Movie 3

Supplemental Movie 4

## Data Availability

The data that support the findings of this study are available from the corresponding author upon reasonable request.
