# Supplementary material for: Multidirectional Planar Motion Transmission on a Single‐Motor Actuated Robot via Microscopic Galumphing
Source: Adv Sci (Weinh). 2023 Dec 14;11(9):2307738. doi: 10.1002/advs.202307738 (PMC10916667; doi:10.1002/advs.202307738)
Supplement: Supplementary file 1 — Supporting Information [file ADVS-11-2307738-s001.pdf]

ADVANCED SCIENCE
Open Access

Supporting Information

for *Adv. Sci.*, DOI 10.1002/advs.202307738

Multidirectional Planar Motion Transmission on a Single-Motor Actuated Robot via Microscopic Galumphing

Lingqi Tang, Chenghao Wang, Songsong Ma, Yao Li and Bing Li**

Supplementary Information

Multidirectional planar motion transmission on a single-motor actuated robot *via* microscopic galumphing

Lingqi Tang†, Chenghao Wang†, Songsong Ma, Yao Li* and Bing Li*
*Corresponding author. Email: liyao2018@hit.edu.cn

Supplementary Information contains:

Supplementary Notes 1-10
Supplementary Figures 1-12
Supplementary Tables 1-4
Supplementary Movies 1-4

Supplementary Text

Supplementary Note 1: Additional Details of the Dynamics Model

The modeling idea is simple but not easy to be implemented due to the various motion status after each collision. Supplementary Figure 3 shows the logic calculation step by step, in which we firstly calculate galumphing motions in the galumphing model. The robot begins with zero speed and jumping height in the both-touching phase, where we can obtain the motor's output force $\vec{F^1}$ in body co-ordinate system $x_1y_1z_1$:

$$\begin{pmatrix} F_x^1 \\ F_y^1 \\ F_z^1 \end{pmatrix} = \begin{pmatrix} \cos q_{tilt} & 0 & \sin q_{tilt} \\ 0 & 1 & 0 \\ -\sin q_{tilt} & 0 & \cos q_{tilt} \end{pmatrix} \begin{pmatrix} F\cos\theta \\ F\sin\theta \\ 0 \end{pmatrix} = \begin{pmatrix} F\cos\theta\cos q_{tilt} \\ F\sin\theta \\ -F\cos\theta\sin q_{tilt} \end{pmatrix} \tag{S1}$$

Also, in the body co-ordinate system, we can obtain the torque around point G, point A, and point B, respectively:

$$\overrightarrow{M_G^1} = \overrightarrow{GO} \times \overrightarrow{F^1} = \begin{pmatrix} -z_{GO}F\sin\theta \\ z_{GO}F\cos\theta\sin q_{tilt} + x_{GO}F\cos\theta\sin q_{tilt} \\ x_{GO}F\sin\theta \end{pmatrix} \tag{S2}$$

$$\overrightarrow{M_A^1} = \overrightarrow{AO} \times \overrightarrow{F^1} + \overrightarrow{AG} \times \vec{g} \cdot m = \begin{pmatrix} -z_{AO}F\sin\theta \\ -\frac{1}{2}Fh\cos\theta + mg \cdot x_{AG} \\ x_{AO}F\sin\theta \end{pmatrix} \tag{S3}$$

$$\overrightarrow{M_B^1} = \overrightarrow{BO} \times \overrightarrow{F^1} + \overrightarrow{BG} \times \vec{g} \cdot m = \begin{pmatrix} -z_{BO}F\sin\theta \\ z_{BO}F\cos\theta\sin q_{tilt} + x_{BO}F\cos\theta\sin q_{tilt} + mg \cdot x_{BG} \\ x_{BO}F\sin\theta \end{pmatrix} \tag{S4}$$

Then, we can calculate the galumphing motion and obtain kinematic parameters as well as the supporting forces during all motion periods as shown in the following:

<u>(1) Both-touching Phase</u>

In this phase, we can obtain the supporting forces N_A and N_B:

$$\begin{cases} N_A = mg - F_z^1 - N_B \\ N_B = \dfrac{x_{AG}}{x_{AB}} mg + \dfrac{M_{Ay}^1}{x_{AB}} \end{cases} \tag{S5}$$

In the both-touching phase, we should alarm for torques in the y-axis and force in the z-axis, which may cause jumps. Specifically, jumping motions of leg A or leg B occur when:

$$action = \begin{cases} A\ jumps\ when \begin{cases} M_{Ay}^1 < 0 \\ M_{By}^1 \le 0\ or \begin{cases} M_G^1 < 0 \\ F_z^1 > 0 \end{cases} \\ F_z^1 \le 0 \end{cases} \\ B\ jumps\ when \begin{cases} M_{Ay}^1 \ge 0 \\ M_{By}^1 < 0\ or \begin{cases} M_G^1 > 0 \\ F_z^1 > 0 \end{cases} \\ F_z^1 \le 0 \end{cases} \end{cases} \tag{S6}$$

<u>(2) Swing Phases</u>

After jumping from the both-touching phase, the robot swings around leg A (for instance), and the rotational motion can be obtained. Motion parameters β_y , w_y ,and q_y denotes angular acceleration, angular speed, and angle around point A in y-axis, respectively. The rotational inertia around leg A in the y-axis is I_{Ay}, we have:

$$\beta_y = \frac{M_{Ay}^1}{I_{Ay}} \quad when\, q_y < 0\, or\, M_{Ay}^1 < 0 \tag{S7}$$

And we can obtain w_y^1 and q_y^1 through integral operation:

$$\begin{cases} w_y = \int \beta_y^1 dt \\ q_y = \iint \beta_y^1 dt^2 \end{cases} \tag{S8}$$

In the swing phase, the motion of COM (point G) is decided by rotation motion around point A. We can obtain the position, velocity, and acceleration of COM as z_G, v_G, and a_G, respectively:

$$\begin{cases} z_G = \overline{GA}\cos(q_2 + q_y) \\ v_G = -w_y \overline{GA}\sin(q_2 + q_y) \\ a_G = -\beta_y \overline{GA}\cos(q_2 + q_y) + w_y \cdot w_y \overline{GA}\cos(q_2 + q_y) \end{cases} \tag{S9}$$

Since only leg A touches the ground, we can obtain the supporting forces as:

$$\begin{cases} N_A = mg - F_z^1 - a_G m \\ N_B = 0 \end{cases} \tag{S10}$$

A-touching can transfer to the air phase if leg A jumps or the both-touching phase if leg B collides with the ground. The judgment condition yields:

$$action = \begin{cases} A\, jumps \quad when\, N_A \le 0 \\ B\, collides\ when\, q_y \ge 0 \end{cases} \tag{S11}$$

(3) Air Phase

In the air phase, the robot moves freely under the actuation of both F and gravity. The motion of the robot can be described as the translational motion of COM and rotational motion around COM, which can be easily obtained:

$$\begin{cases} a_z = \dfrac{(F_z^1 - mg)}{m} \\ v_z = \int a_z dt \\ z_G = \iint a_z dt^2 \end{cases} \tag{S12}$$

$$\begin{cases} \beta_y = \dfrac{M_{Gy}^1}{I_{Gy}} \\ w_y = \int \beta_y dt \\ q_y = \iint \beta_y dt^2 \end{cases} \tag{S13}$$

And we should alarm for the position of the two legs, corresponding to the condition of collision of the legs:

$$action = \begin{cases} A\, collides\ when\ z_G - \overline{GA}\cos(q_2 + q_y) \le 0 \\ B\, collides\ when\ z_G - \overline{GB}\cos(q_3 - q_y) \le 0 \end{cases} \tag{S14}$$

Finally, we can calculate the planer motion model.

Supplementary Note 2: Motor output force test

To estimate the motor output force accurately, we built a look-up table (LUT) based on the experimental data. We fixed the motor with a 6-axis force sensor (Nano17, ATI), shown in Supplementary Figure 3A. We fixed the supply voltages equally from 0.7 V to 3.3 V with 0.2 V increments. When the motor was spinning stably under constant voltage, the output forces in the motor's plane were recorded at 30 kHz. Then we calculated the main amplitude of forces in the x-axis and y-axis as the output force under certain voltages. Each test was repeated three times.

As shown in Supplementary Figure 4A, the LUT was a nonlinear fit ($y = y_0+A(|x-x_c|^P)$, *R square 0.963*) of the testing results, with parameters shown in Supplementary Table 2. We also present the relationship between applied voltage and motor speed as well as centrifugal force, as shown in Supplementary Figure 4B. The motor's speed varied from 319 rad/s to 1363 rad/s, corresponding lowest (0.7 V) and highest (3.3V) voltages. The relationship between output force and motor speed is not quadratic, indicating that the output force was not only contributed by centrifugal force but may also contain frictional forces from the bearing and the brush. But the force and speed still monotonically increased as w increased. Thus, we can simply change the motor output force and speed via changing the input voltage.

Supplementary Note 3: Details of galumphing and planner motion tests

The testing setups for observing galumphing motion and planner motion are shown in Supplementary Figure 5. The observed video of galumphing motion was processed frame by frame using corresponding software (Pronatalist). The results showed that the observed galumphing gait is similar to the modeling results, as shown in Supplementary Figure 6. Although the jumping heights (100-150 μm) of leg A are different from the modeling results (50–100 μm), we can still find that leg A always lands after the leaping of leg B. Thus, the key factor of ERDMT, the timing of ground-touching of leg A, was similar to the modeling results.

Supplementary Note 4: Design methods of GASR

The development process of GASR was instructed by both the dynamics model and practical testing. We kept the two-leg structural configuration in Section 2 but employed a smaller battery and a smaller printed circuit board (PCB), on which we selected a proper position of the motor. Thus, the main parameter is the distance between COM and leg A (GA_x in the model), which should not be too small or too large. Regarding GASR with total length of 25 mm, if GA_x is smaller than 1 mm, the torque of gravity that resists the galumphing motion would be too small, causing unstable galumphing. However, when GA_x becomes larger than 5 mm, the slipping mode would take up too much of the optional motor speed range, squeezing the critical phase (straight forward) to the unstable zone. GA_x was set to 4.5 mm, and then the locations of the motor and the battery were obtained.

As shown in Supplementary Figure 7A, GASR consists of a PCB, a motor as the front leg, a 10 mAh Li-Po battery, and a Kapton scrap as the hind leg. The materials are light and cheap under mass-production, as shown in Supplementary Table 3. To be noted, this motor is a modified version of the previous motor (0720, LEADER) with reduced eccentric mass to produce smaller force.

In the upper computer, three excitation voltages (PWM voltage signal) were preset for the three motion modes above. Thereafter, GASR can be manually controlled by a human effectively. The command of motion is transferred to the robot via Bluetooth communication. We employed nRF52832 from Nordic as both the main control chip and Bluetooth communication chip, which has a compact footprint (BGA 3 mm×3 mm). A H-bridge-based motor driver, DRV8833 from TI, was used to drive the motor. Our PCB was designed to be small (8 mm × 23 mm) and light (0.45 g), with all components mounted on the front side. Thus, the motor can be mounted to the backside without unexpected tilting from the main body. The schematic diagram is shown in Supplementary Figure 7B.

Supplementary Note 5: Demonstration of making a simple ERDMT-based robot in 10 mins

ERDMT can be replicated not only on robots with compliant legs, but also on other configurations. Here, we demonstrate the implementation of a simple ERDMT-based prototype using material from everyday life. As shown in Supplementary Figure 8A, this external-powered robot consists of three parts: an eccentric motor (0720, LEADER), a medium-sized clamp, and a postcard with a thickness of 0.5 mm. This structure configuration also tilted the eccentric motor, but has a rigid hind leg.

As shown in Supplementary Figure 8, the prototype moved along a quasi-straight line under a constant voltage of 1.36 V, and turned left or right under lower (1.17 V) and higher (1.50 V) voltages, respectively. According to the recorded videos, we can roughly estimate the forward speed as ~21 mm/s (0.3 BL/s) and the steering speed as ~48°/s. What's more, similar to GASR, this prototype has also revealed an initial defection angle (41°) and a self-misalignment angle (31°) with respect to the forward moving direction.

Supplementary Note 6: Comparison of single motor actuated robots

Herein, we specifically compared GASR with previous single actuator based robots: 1STAR, PISCES, Simobot, BFFSPR, TSIGS robot, and SUTD Robot. 1STAR is a single servo motor actuated hexapod robot, utilizing a disparity of different novel gaits, which were generated by ingenious transmission mechanism design and accurate motor phase control. However, 1STAR was lack of agility due to the locomotion principle and cumbersome transmission structures. PISCES, a piezoelectric-actuated walking robot, can perform both forward and spot-turning movements with different walking modes modulated by varying actuation frequencies. However, the miniaturization of PISCES is constrained by its complex high-voltage excitation circuitry. SimoBot is actuated by an eccentric motor, performing circular trajectories. Then, the straight trajectory can be achieved by splicing multiple small curves. Therefore, complicated path plans and control methods are needed. The BFFSPR, TSIGS, and SUTU robots are all soft tethered robots. Different excitation frequency produces different bending modes of the piezoelectric plate or dielectric elastomer, generating different gaits. But the complex excitation increases the difficulty of control. In this work, GASR performs forward crawling, turning, and spot steering motions under constant voltages. The multimodal locomotion strategy assures the agility of the robot. The simple structure and excitation significantly cut down the mass (1.2 g) and cost (3.9 $) of the robot. What’s more, GASR revealed agile and precise planar movements, indicating the high effectiveness of ERDMT.

Supplementary Note 7: The relationship between the weight of the robot's body and the actuation

Based on the dynamic model, we studied the impact on the motion when varying the actuation force. Meanwhile, other design parameters were set as in Supplementary Table 1. As shown in Supplementary Figure 9, when F_{0max}/mg is lower than 2.6, it is impossible to find a critical motor speed within the operational range. Thus, the robot can not actuate eccentric-rotation-dependent multidirectional transmission (ERDMT). When F_{0max}/mg equals 3.5, the critical motor speed exists in the operational range. However, if F_{0max}/mg is larger than 4.3, the curve becomes steeper, and the motion robustness decreases. Thus, F_{0max}/mg is preferred to lie in the range 3.5–4.3.

Supplementary Note 8: Structural factors in relation to the motion of GASR

Based on the dynamics model, we investigated the impacts on the planar motion transmission when varying different parameters, e.g., motor tilting angle, leg stiffness (similar to collision coefficient), distance between COM and the two legs, and frictional coefficient. The tilting angle influences the critical motor speed and changes the amplitudes of angular velocities. As in Supplementary Figure 10A, the motion curve mainly shifted to the left when the tilting angle q_{tilt} varied from 5° to 25°. The tilting angles of 5° and 10° are abandoned because the critical motor speed exceeds the motor's operational limit (1000 rad/s). When the tilting angle is 25°, the motion has multiple critical motor speeds, whereas the motion robustness decreases due to the increased slopes. The collision coefficient influences the rhythmic galumphing motion. In Supplementary Figure 10B, C, and D, a larger collision coefficient led to unexpected bouncing, affecting motion stability. The ratio between GA_x and AB_x is another crucial design parameter indicating the position of COM and the two legs. In Supplementary Figure 10E, when GA_x/AB_x is 0.05, the COM is too close to Leg A, and then a smaller gravitational torque is applied around Leg A. Thereafter, the actuation force generates a larger swing, causing unstable motion. However, when GA_x/AB_x is larger than 0.25, the gravitational torque becomes too large, affecting the motion robustness. As shown in Supplementary Figure 10F, the frictional coefficient has minor impact on motion than that of other parameters.

Supplementary Note 9: The planar movement of the robot is a distinct type of motion analogous to the galumphing of seals.

The sequences of the terrestrial galumphing motion of seals were studied. Spinal flexion was adopted to help seals quickly lift their bodies into the air, as shown in the videos: https://tenor.com/zh-CN/view/seal-running-away-cute-weird-animal-gif-17851078, https://www.tiktok.com/@integralfun/video/7204709604771515694. Indeed, in some cases, the fore flippers contribute to movements partially as an anchor, while some other seals were observed to move solely by spinal flexion without using the fore flippers [1]. Although the actuation mechanisms differ between the seals and the robot, they move forward by alternately lifting their fore and hind bodies off the ground. Both the seals and the robot have wave-like motions [1] [2]. Moreover, duty factors of leg A's air-phase in Galumphing Mode 2 & 3 (Figure 2) were 49.7% and 54.2%, respectively. The mean duty factors of the forebody of the wild harbor and gray seals [1] were 54.1% and 53.8% of the cycle, respectively. The duty factor of the forebody of the robot is close to that of the seal. Thus, the planar movement of the robot can be analogous to the galumphing of seals.

Supplementary Note 10: The influences of slope and substrate roughness on crawling

The robot has the ability to climb stably on different slopes. We tested the slope climbing ability from 18° to 25°. As shown in Supplementary Figure 12, the robot succeeded in climbing a slope of 18°, 20°, and 22°, but failed to climb a 25° slope. When going up on 25° slope, the robot moved laterally because of the actuation limit. Moreover, we tested the robot's downhill crawling ability on slopes from 15° to 32°. The robot could perform downhill crawling when the angle was less than 30°, but slipped off on the 32° slope.

In Supplementary Movie 2, the robot was tested on different substrates, such as aluminum alloy plates, paper boards, and foam boards. We assume the lateral error of a straight forward movement should be smaller than 5%. In Supplementary Figure 13, the robot can perform straight forward crawling and spot steering on a P320 Grade sandpaper. However, it was stuck on a P120 Grade sandpaper and lost motion stability on a P240 Grade sandpaper. Thus, the robot can currently function well on surface with mild roughness (P320 Grade sandpaper, aluminum plate, foam board, etc.).

Supplementary figures

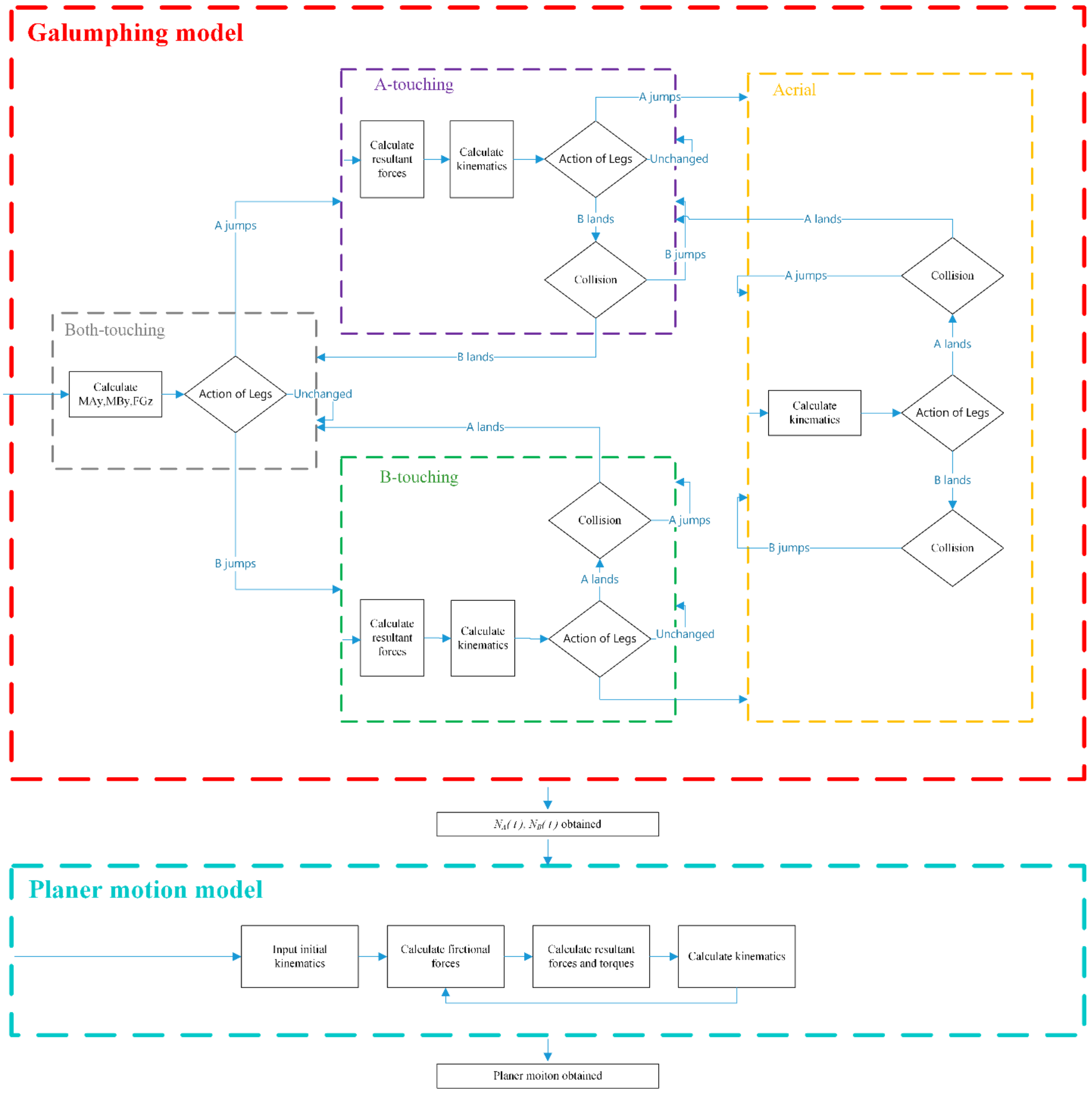

Supplementary Figure 1: Illustrative block diagram of the dynamics model implementation. The overall dynamic model was divided into two-DOF galumphing model and three-DOF planer motion model. Once the calculation of galumphing model is completed, the supporting forces were imported into planer motion model to obtain planer motions.

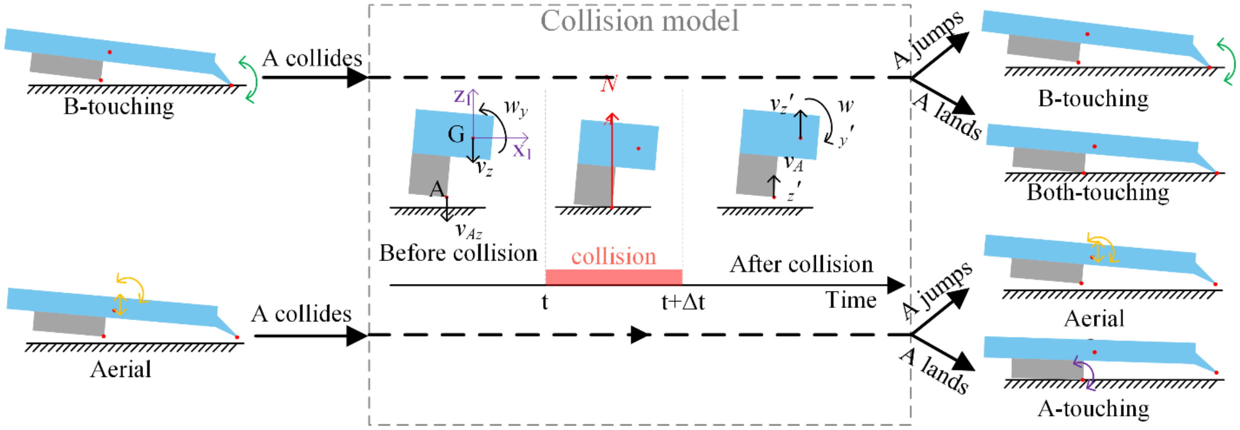

Supplementary Figure 2: Schematic of the collision calculation process of leg A. Once leg A collides with the ground, all motion parameters can be calculated based on the conservation of angular momentum around leg A. Assuming the collision time is Δt, we can obtain the collision supporting force N_A. Thereafter, we can obtain the motion status after the collision, which depends on the speed of leg A and the status before the collision.

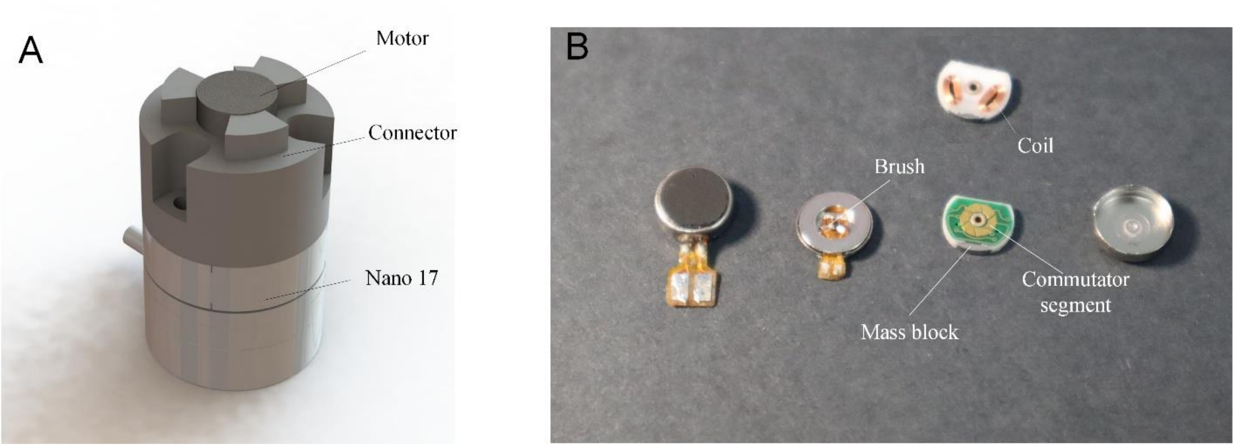

Supplementary Figure 3: Eccentric motor speed and centrifugal force calibration experiment. (A) Placement of the motor on force sensor Nano 17. **(B)** Disassembled motor showing that the motor speed can be measured thanks to the commutator.

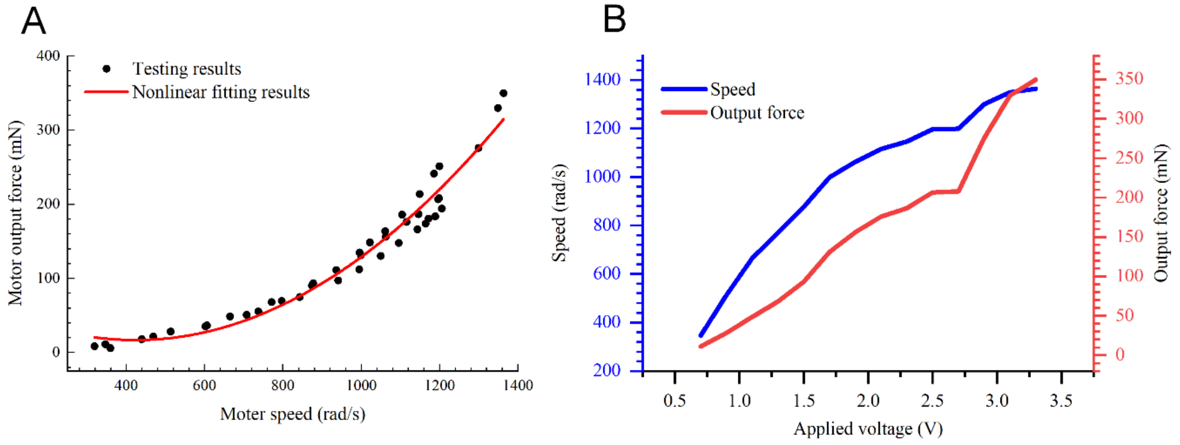

Supplementary Figure 4: Output force and speed test of the eccentric motor. (A) Relationship between motor force and motor speed. The nonlinear fitting is utilized as a look-up table in the modeling section. **(B)** Under different applied voltages, the motor exhibits different speed and force performance.

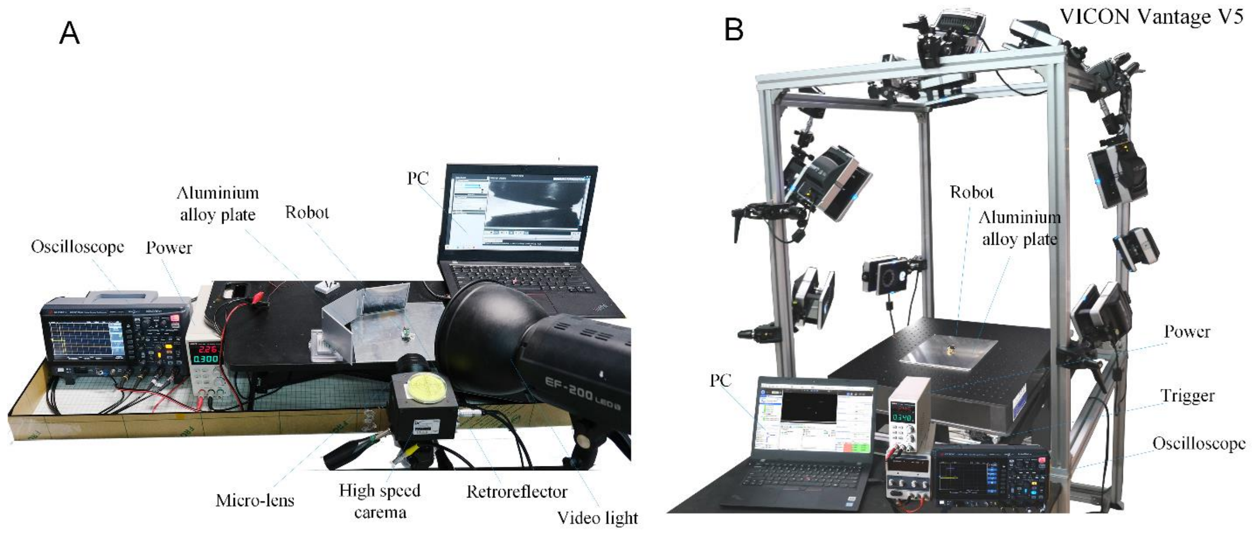

Supplementary Figure 5: The testing setups for observing galumphing motion and planner motion. (A) Observe the galumphing motion in micro-scale. **(B)** Planner motion test set up.

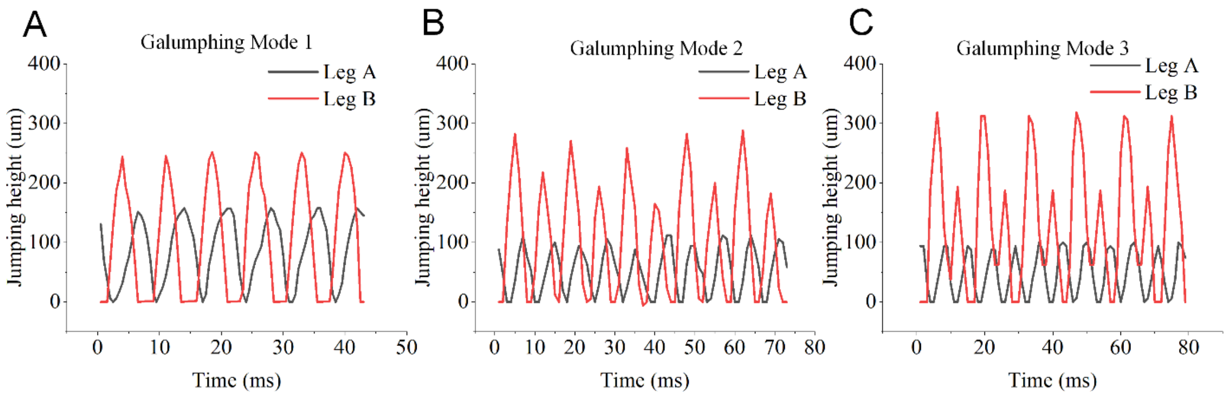

Supplementary Figure 6: Observed jumping heights of the two legs in the different galumphing modes. (A) Galumphing mode 1. **(B)** Galumphing mode 2. **(C)** Galumphing mode 3.

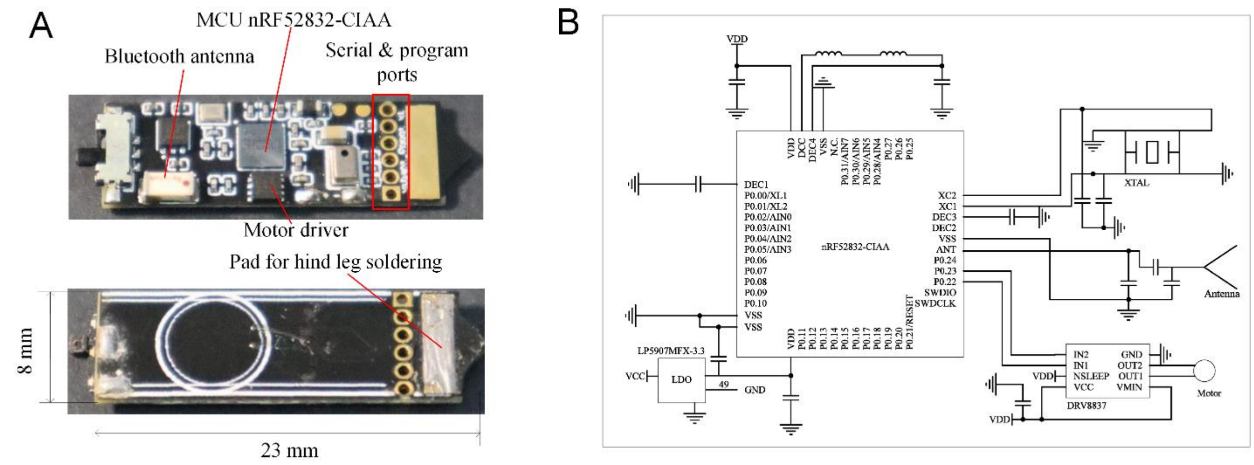

Supplementary Figure 7: PCB design for GASR. (A) Photos of the PCB from both sizes. **(B)** Schematic diagram of the PCB.

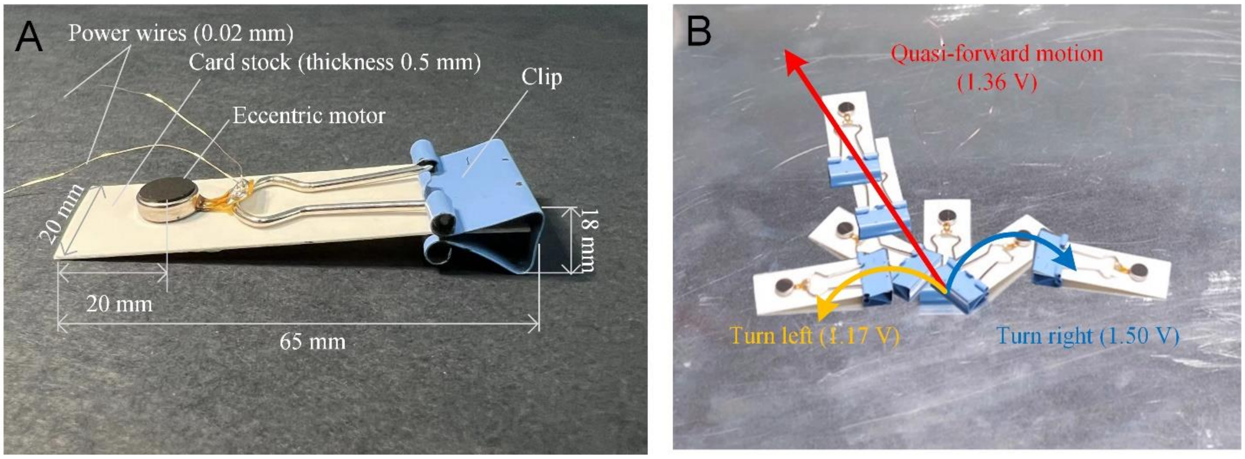

Supplementary Figure 8: A simple prototype revealed ERDMT clearly. Common materials were used to build the prototype. The front leg (card stock) can be assumed as an absorber, while the hind leg (clip) is rigid.

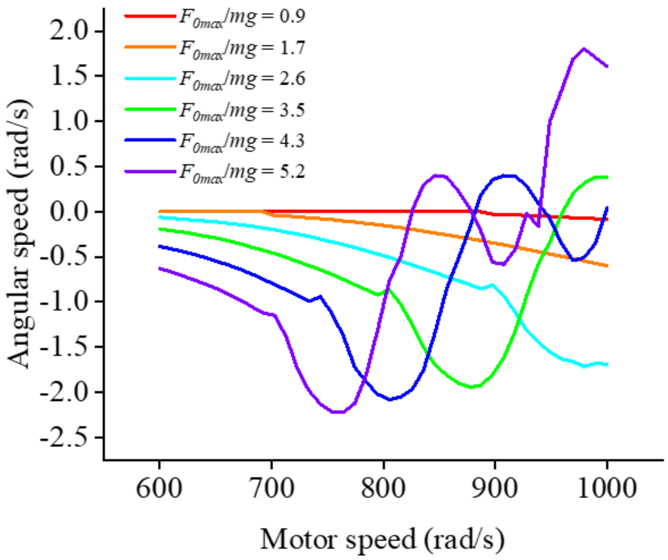

Supplementary Figure 9. The ratio between actuation and weight significantly affects the motion.

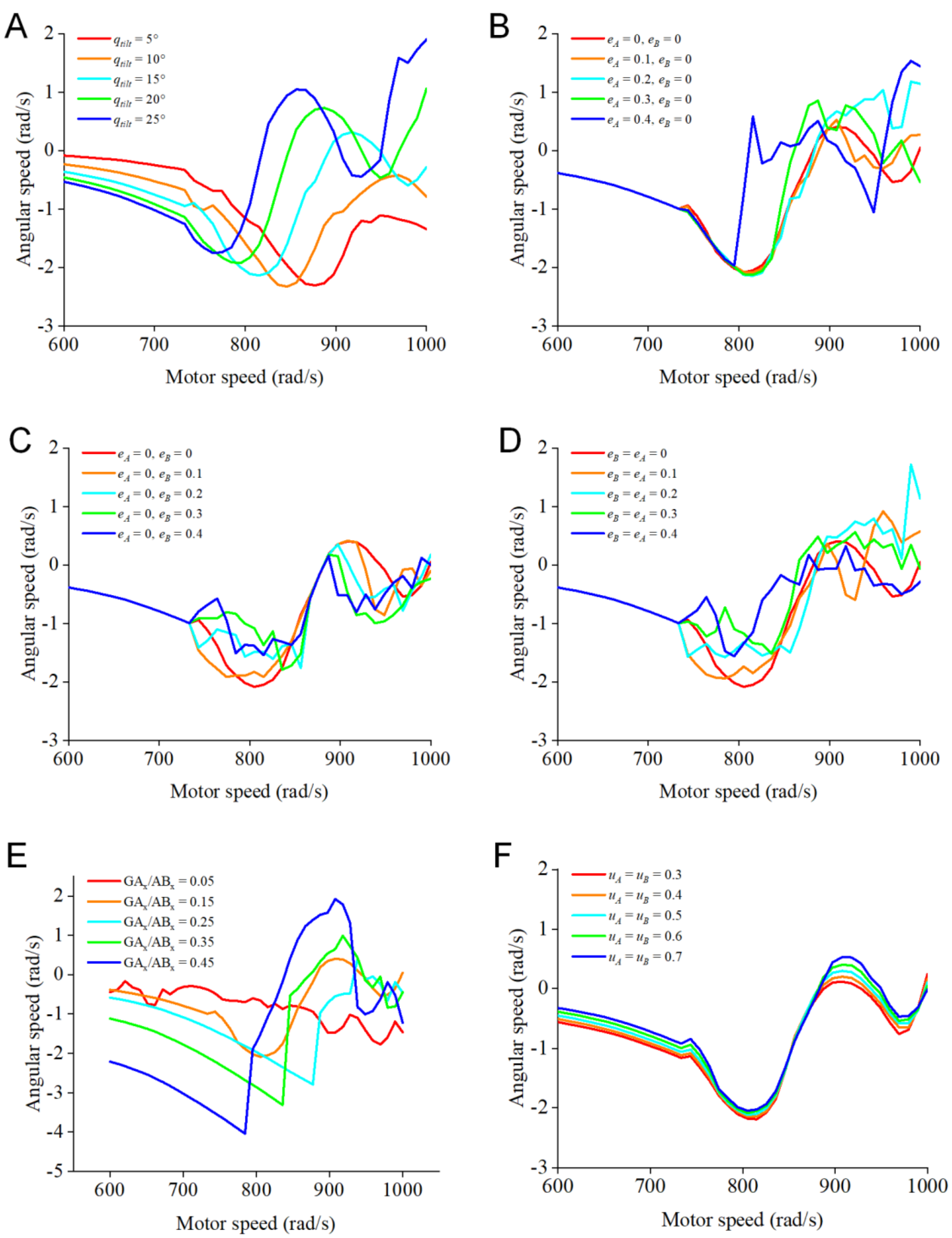

Supplementary Figure 10. Impacts on motion from different design parameters.

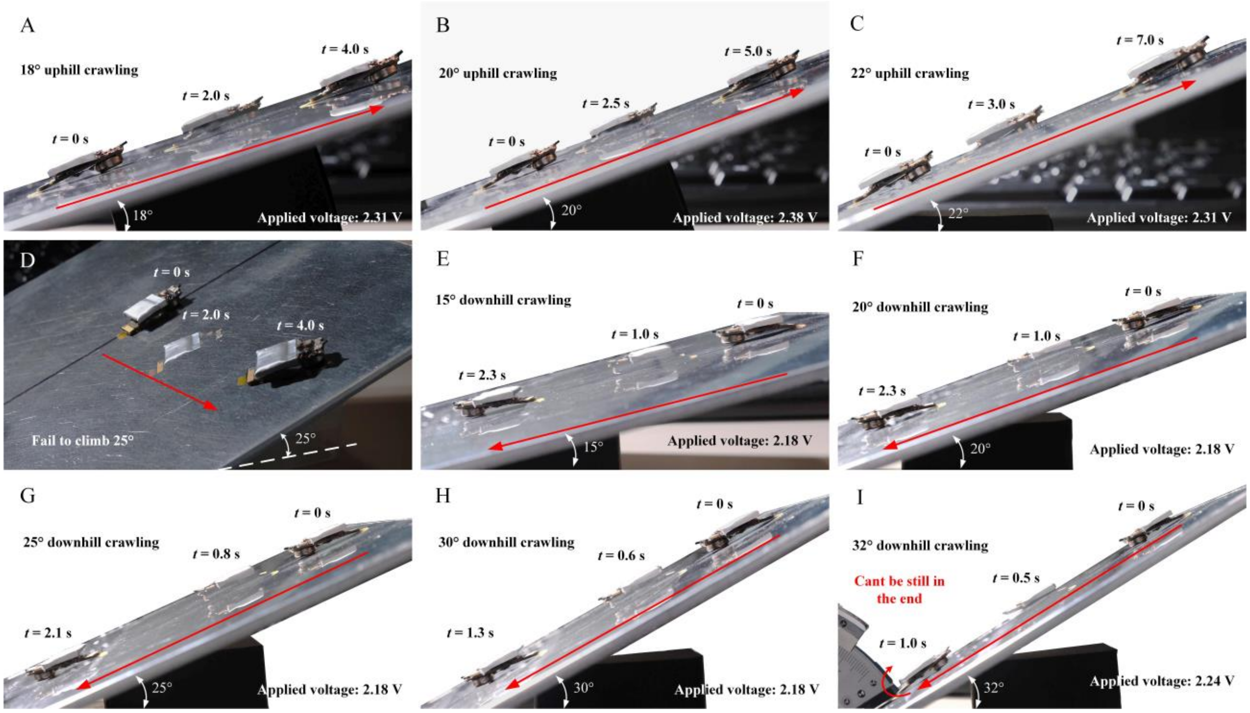

Supplementary Figure 11. GASR going up and down slopes. The robot succeeded in climbing slopes of 18° **(A)**, 20° **(B)**, and 22° **(C)** but failed on 25° slope **(D)**, performing a lateral movement instead. The robot can go downhill a slope of 15° **(E)**, 20° **(F)**, 25° **(G)**, and 30° **(H)**. **(I)** The robot quickly moved down a 32° slope but slipped and dropped off the plate.

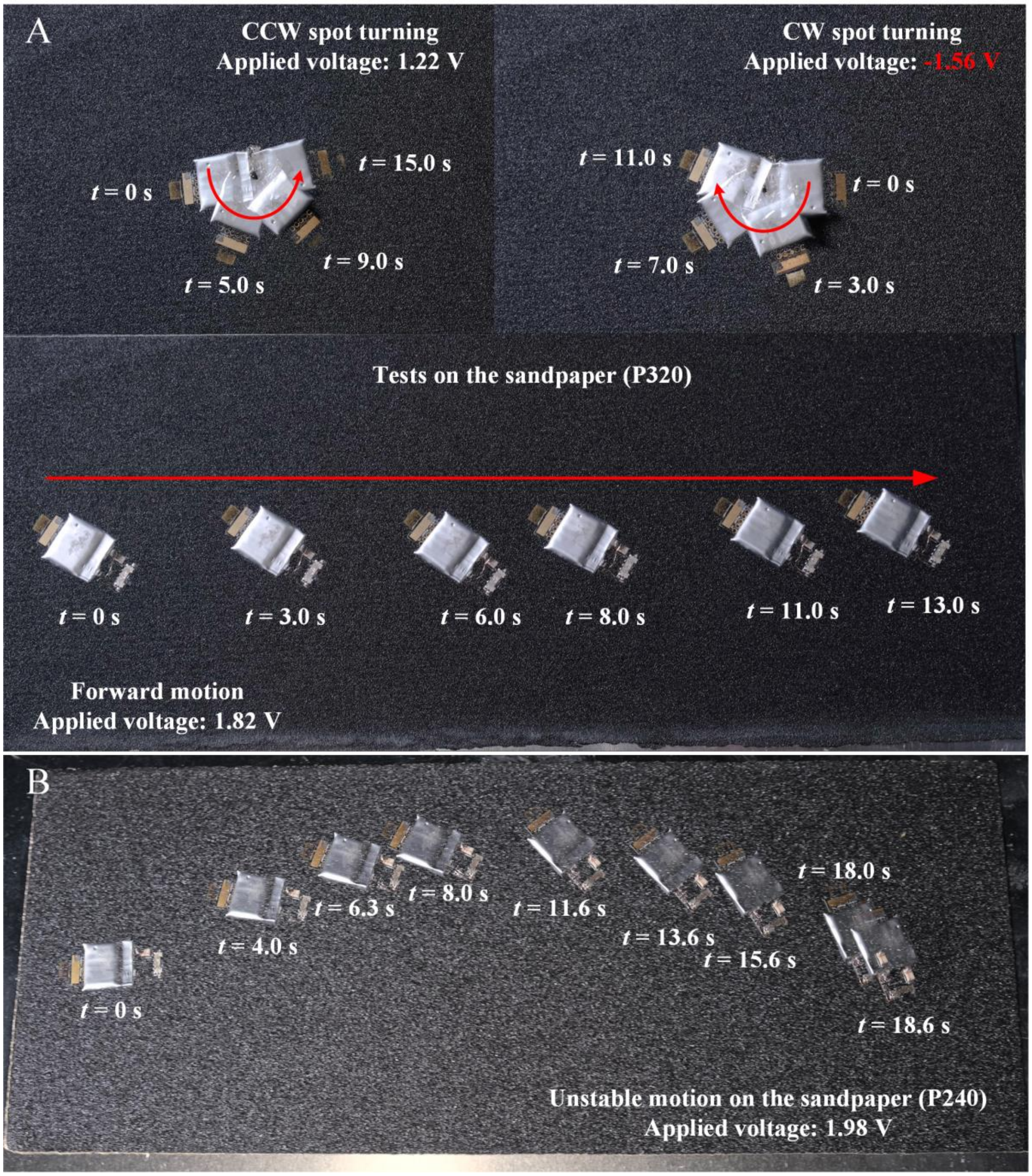

Supplementary Figure 12. The influences of the substrate roughness. (A) Straightforward crawling and steering in place on a P320 Grade sandpaper. **(B)** Unstable motion on a P240 Grade sandpaper.

Supplementary tables

Supplementary Table 1. Parameters of the dynamics model. The calculation step of the iterative model is set to 0.1 rad out of the rotor position. The frictional coefficients were obtained by practical tests on different slopes. And the collision recovery coefficients were set to zero due to the compliant design of the two legs.

Parameter description	Symbol	Value	Unit
Calculation step	$d\theta$	0.1	rad
Motor tilt angle	q_{tilt}	16	deg
Mass	m	3.1	g
Motor height	h	3	mm
Motor outer radius	r	3.5	mm
Length AB in x_0-axis	AB_x	20	mm
Length GA in x_0-axis	GA_x	3	mm
Moment of inertia around Point G in the z-axis	I_{Gz}	193.87	gmm^2
Moment of inertia around Point G in the y-axis	I_{Gy}	89.43	gmm^2
Moment of inertia around Point B in the y-axis	I_{By}	1069.81	gmm^2
Moment of inertia around Point A in the y-axis	I_{Ay}	141.14	gmm^2
Moment of inertia around Point A in the x-axis	I_{Ax}	138.85	gmm^2
Friction coefficient of leg B	u_B	0.62	-
Friction coefficient of leg A	u_A	0.55	-
Collision recovery coefficient of Leg A	e_A	0	-
Collision recovery coefficient of Leg B	e_B	0	-

Supplementary Table 2. Nonlinear fitting parameters of motor output force. To estimate the motor output force accurately, we built a look-up table (LUT) based on the experimental data. As shown in Supplementary Figure 4A, the LUT was a nonlinear fit ($y = y_0+A(|x-x_c|^P)$, *R square 0.963*) of the testing results, with parameters shown in Supplementary Table 2.

	Value	Standard Error
y_0	16.75425	5.89048
x_c	423.55055	42.19814
A	3.37306E-4	3.25776E-5
P	2	0

Supplementary Table 3. Weight and Cost Budget of GASR.

Component	Weight [g]	Cost [$]
Motor	0.37	0.37
Battery	0.39	1.31
PCB*	0.45	2.24
Kapton	0.03	-
Total	1.24	3.92

* PCB plate manufacturing and surface mounted technology (SMT) costs are evaluated for mass production.

Supplementary Table 4. Small-scale planer crawling robots overview.

		Robot	Mass [g]	Length [mm]	Motion type	Actuator	Excitation	Power Consumption [mW]	Continues forward motion	Spot turn
Untethered	Multiple actuators	RoACH [3]	2.4	30	ALS[e)]	2 SMAs[a)]	Constant	~1660[h)]	●	●
		MinRAR [4]	27.5	55	ALS	12 PCs	Sinusoidal wave	2350	●	●
		HAMR-F [5]	2.8	45	ALS	8 PCs[b)]	Sinusoidal wave	395	●	●
		Δ-Type Robot [6]	45	55	VS	3 PCs	Sinusoidal wave	< 1000	●	●
		Kilobot [7]	8	33	VS[f)]	2 EMs[c)]	Constant	~20[h)]	●	●
		UW robot [8]	2.8	20	VS	2 EMs	Constant	33	●	●
		Amphibious Robot [9]	35	95	ALS, VS	2 EMs	Constant	846	●	●
	Single actuator	1STAR [10]	80	120	ALS	1 DC motor	Square wave	178	●	×
		PISCES [11]	21	90	VS	1 PC	Sinusoidal wave	>10000[h)]	●	●
		SimoBot [12]	4.76	20	VS	1 EM	Square wave	44	×	●
		**GASR**	**1.2**	**25**	**GA[g)], VS**	**1 EM**	**Constant**	**62**	●	●
Tethered		BFFSPR [13]	0.058	10	GA	1 PC	Square wave	0.3 [h)]	●	×
		TSIGS robot [14]	0.067	30	GA	1 PC	Sinusoidal wave	0.343	●	×
		SUTU robot [15]	0.51	87	GA	1 DE[d)]	Sinusoidal wave	0.64 [h)]	●	×

[a)]SMA: Shape memory alloy; [b)]PC: Piezoelectric Ceramic; [c)]EM: Eccentric rotating mass vibration Motors; [d)]DE: dielectric elastomer. [e)]ALS: Active leg-based slipping; [f)]VS: Vibration-based slipping; [g)]GA: Galumphing; [h)]Some parameters are estimated according to the literature.

Supplementary Movie 1. Galumphing motion observation. Forward galumphing motions were analyzed using high-speed microscopic imaging. The results showed that the observed galumphing gait is similar to the modeling results, as shown in Supplementary Figure 6.

Supplementary Movie 2. GASR: performance testing. The video shows GASR performing on-the-spot turning, straightforward crawling, and forward left/right crawling at different voltages. Moreover, the video demonstrates GASR climbing a 15° slope and crawling on different substrates.

Supplementary Movie 3. Manually controlled locomotion of GASR. The video shows that GASR follows a squared shape, a 'z' shape, and can crawl in a narrow space.

Supplementary Movie 4. Making a simple ERDMT-based robot in 10 mins. The video demonstrates the implementation of a simple ERDMT-based prototype using material from everyday life.

Reference

[1] J. N. Garrett and F. E. Fish, "Kinematics of terrestrial locomotion in harbor seals and gray seals: Importance of spinal flexion by amphibious phocids," *Mar. Mammal Sci.*, vol. 31, no. 2, pp. 459–478, 2015, doi: 10.1111/mms.12170.

[2] S. Kerr and S. J. Kerr, "The Biomechanical Energetics of Terrestrial Locomotion in The Biomechanical Energetics of Terrestrial Locomotion in California Sea Lions (Zalophus californianus): Efficiency of California Sea Lions (Zalophus californianus): Efficiency of Quadrupedal Gallo," 2021. [Online]. Available: https://digitalcommons.wcupa.edu/all_theses/208

[3] A. M. Hoover, E. Steltz, and R. S. Fearing, "RoACH: An autonomous 2.4g crawling hexapod robot," in *2008 IEEE/RSJ International Conference on Intelligent Robots and Systems, IROS*, 2008, pp. 26–33. doi: 10.1109/IROS.2008.4651149.

[4] S. A. Rios, A. J. Fleming, and Y. K. Yong, "Monolithic Piezoelectric Insect with Resonance Walking," *IEEE/ASME Trans. Mechatronics*, vol. 23, no. 2, pp. 524–530, 2018, doi: 10.1109/TMECH.2018.2792618.

[5] B. Goldberg *et al.*, "Power and Control Autonomy for High-Speed Locomotion with an Insect-Scale Legged Robot," *IEEE Robot. Autom. Lett.*, vol. 3, no. 2, pp. 987–993, 2018, doi: 10.1109/LRA.2018.2793355.

[6] J. Li, J. Deng, Y. Liu, S. Member, S. Zhang, and K. Li, "Development of a Planar Tripodal Piezoelectric Robot With a Compact Ring Structure," *IEEE/ASME Trans. Mechatronics*, vol. PP, no. c, pp. 1–12, 2022, doi: 10.1109/TMECH.2022.3148086.

[7] M. Rubenstein, C. Ahler, N. Hoff, A. Cabrera, and R. Nagpal, "Kilobot: A low cost robot with scalable operations designed for collective behaviors," *Rob. Auton. Syst.*, vol. 62, no. 7, pp. 966–975, 2014, doi: 10.1016/j.robot.2013.08.006.

[8] V. Iyer, A. Najafi, J. James, S. Fuller, and S. Gollakota, "Wireless steerable vision for live insects and insect-scale robots," *Sci. Robot.*, vol. 5, no. 44, pp. 1–13, 2020, doi: 10.1126/scirobotics.abb0839.

[9] D. Wang *et al.*, "Miniature Amphibious Robot Actuated by Rigid-Flexible Hybrid Vibration Modules," *Adv. Sci.*, vol. 9, no. 29, pp. 1–12, 2022, doi: 10.1002/advs.202203054.

[10] D. Zarrouk and R. S. Fearing, “Controlled in-plane locomotion of a hexapod using a single actuator,” *IEEE Trans. Robot.*, vol. 31, no. 1, pp. 157–167, 2015, doi: 10.1109/TRO.2014.2382981.

[11] H. H. Hariri, G. S. Soh, S. Foong, and K. L. Wood, “A highly manoeuvrable and untethered under-actuated legged piezoelectric miniature robot,” in *Proceedings of the ASME Design Engineering Technical Conference*, 2019, vol. 5B-2019, pp. 1–10. doi: 10.1115/DETC2019-97353.

[12] Y. Zhang, R. Zhu, J. Wu, and H. Wang, “SimoBot: An Underactuated Miniature Robot Driven by a Single Motor,” *IEEE/ASME Trans. Mechatronics*, vol. 27, no. 6, pp. 1–12, 2022, doi: 10.1109/TMECH.2022.3189218.

[13] E. Chen *et al.*, “Bio-Mimic, Fast-Moving, and Flippable Soft Piezoelectric Robots,” *Adv. Sci.*, 2023, doi: 10.1002/advs.202300673.

[14] Y. Wu *et al.*, “Insect-scale fast moving and ultrarobust soft robot,” *Sci. Robot.*, vol. 4, no. 32, 2019, doi: 10.1126/scirobotics.aax1594.

[15] D. Wang *et al.*, “Dexterous electrical-driven soft robots with reconfigurable chiral-lattice foot design,” *Nat. Commun.*, vol. 14, no. 1, 2023, doi: 10.1038/s41467-023-40626-x.
